# Antibody and T Cell Responses in Reciprocal Prime-Boost Studies with Full-Length and Truncated Merozoite Surface Protein 1–42 Vaccines

**DOI:** 10.1371/journal.pone.0075939

**Published:** 2013-09-30

**Authors:** Kae Pusic, Danielle Clements, Sophie Kobuch, George Hui

**Affiliations:** University of Hawaii, School of Medicine, Department of Tropical Medicine, Honolulu, Hawaii, United States of America; Federal University of São Paulo, Brazil

## Abstract

The *P. falciparum* Merozoite Surface Protein 1–42 (MSP1-42) is one of the most studied malaria subunit vaccine candidates. The N-terminal fragment of MSP1-42, MSP1-33, is primarily composed of allelic sequences, and has been shown to possess T helper epitopes that influence protective antibody responses toward the C-terminal region, MSP1-19. A truncated MSP1-42 vaccine, Construct 33-I, consisting of exclusively conserved T epitope regions of MSP1-33 expressed in tandem with MSP1-19, was previously shown to be a more effective immunogen than the full-length MSP1-42 vaccine. Here, by way of reciprocal priming/boosting immunization regimens, we studied the immunogenicity of Construct 33-I in the context of recognition by immune responses induced by the full-length native MSP1-42 protein, in order to gauge the effects of pre- and post-exposures to MSP1-42 on vaccine induced responses. Judging by immune responsiveness, antibody and T cell responses, Construct 33-I was effective as the priming antigen followed by full-length MSP1-42 boosting, as well as the boosting antigen following full-length MSP1-42 priming. In particular, Construct 33-I priming elicited the broadest responsiveness in immunized animals subsequently exposed to MSP1-42. Moreover, Construct 33-I, with its conserved MSP1-33 specific T cell epitopes, was equally well recognized by homologous and heterologous allelic forms of MSP1-42. Serum antibodies raised against Construct 33-I efficiently inhibited the growth of parasites carrying the heterologous MSP1-42 allele. These results suggest that Construct 33-I maintains and/or enhances its immunogenicity in an allelic or strain transcending fashion when deployed in populations having prior or subsequent exposures to native MSP1-42s.

## Introduction

Efforts to develop a blood-stage malaria vaccine have focused on a number of antigens [1], [2], among them *P. falciparum* Merozoite Surface Protein 1 (MSP1). MSP1 is one of the major proteins on the surface of invading merozoites, and it undergoes two sequential proteolytic cleavages during blood-stage development [3], [4]. The first cleavage forms four fragments; subsequently, the C-terminal fragment, Merozoite Surface Protein 1–42 (MSP1-42), is further cleaved to yield a 33 kDa (MSP1-33) and a 19 kDa fragment (MSP1-19) [4]. During merozoite invasion, the C-terminal MSP1-19, which is largely conserved across allelic forms [5], remains associated with the merozoite surface membrane and is carried into the erythrocyte. On the other hand, MSP1-33, which is comprised of mostly dimorphic allelic sequences, is released into the blood plasma [6].

Both MSP1-42 and MSP1-19 have shown potential as subunit vaccines in rodent and monkey models [7]–[11]. Passive transfers of anti-MSP1-42 or anti-MSP1-19 monoclonal antibodies have been found to protect against malaria [12], [13], and appear to do so via inhibition of merozoite invasion and/or by opsonization [14], [15]. Anti-MSP1-42/MSP1-19 antibodies have also been shown to correlate with naturally acquired immunity in several epidemiological studies [16]–[20].

Studies on MSP1-33 have identified a number of T cell epitopes [21]–[23]. It has been suggested that these T cell epitopes provide cognate helper function for the production of anti-MSP1-19 antibody responses [22]–[27]. In a recent study, we examined the potential role of MSP1-33 specific T cell epitopes in influencing the immunogenicity of MSP1-42 based vaccines [28]. Accordingly, nine truncated MSP1-42 recombinant proteins, each with a different combination of MSP1-33 specific T cell epitopes linked to MSP1-19, were assessed for immunogenicity. The results demonstrated that different T cell helper epitopes on MSP1-33 have positive or negative effects on the induction of inhibitory antibodies. The study provided insights into how anti-MSP1-19 antibody responses can be modulated during vaccination and natural infections [28]. The same study also identified two truncated MSP1-42 constructs, Construct 33-D and Construct 33-I, that shows greater vaccine potential than the full-length native MSP1-42 [28]. Construct 33-D is comprised of both allelic and conserved regions of MSP1-33; whereas, Construct 33-I consists of only conserved regions of MSP1-33 fused in tandem with MSP1-19. This truncated Construct 33-I induces anti-MSP1-19 antibodies that have more potent *in vitro* parasite growth inhibitory activities than those induced by Construct 33-D or by the full length MSP1-42 [28]. Moreover, Construct 33-I, because of its make up of conserved sequences of MSP1-33, has the potential to elicit strain transcending immunity against heterologous parasite strains. Based on these attributes, Construct 33-I is thus more attractive than Construct 33-D as a malaria vaccine and is the focus of our present study.

Since Construct 33-I is an artificially truncated MSP1-42 protein based on tandem fusion of 6 conserved sequence blocks of MSP1-33 to MSP1-19 [28], it is important from a vaccine development and deployment point of view to evaluate its immunogenicity in the context of recognition by immune responses to the full-length native MSP1-42. To this end, we evaluated the antibody and T cell immunogenicity of Construct 33-I when given as a priming or boosting immunogen in outbred mice that were previously primed or subsequently re-challenged with full-length MSP1-42 (Table 1). Furthermore, the effects of dimorphic allelism of MSP1-33 on the immunogenicity of Construct I were examined using the same prime/boost regimen in order to gauge its potential to elicit allele or strain transcending responses in the context of the full length MSP1-42 alleles.

**Table 1:** Summary of Prime/Boost Immunizations with Truncated MSP1-42 (Construct 33-I) and Full Length MSP1-42s.

Immunization Regimens	Antigen used for Priming	Antigen used for Boosting
1	Construct 33-I	Construct 33-I
2	Construct 33-I	MSP1-42
3	MSP1-42	Construct 33-I
4	Construct 33-I	MSP1-42 (FVO)
5 (control)	MSP1-42	MSP1-42
6 (control)	MSP1-42	MSP1-42 (FVO)

## Materials and Methods

### Ethics Statement

Use of mice was approved by the University of Hawaii’s Institutional Animal Assurance (A3423-10), protocol number (08-389-6).

### Mouse Strains

Outbred Swiss Webster (SW) mice (female, 6–8 weeks old) were obtained from Charles River Laboratory (Wilmington, MA). Cr: NIH(S)-nu/nu mice (NIH Swiss background, female, 6 weeks old) were obtained from NCI Frederick (Frederick, Maryland).

### Recombinant Truncated MSP1-42 and Full-length MSP1-42 Proteins

**Figure 1:**
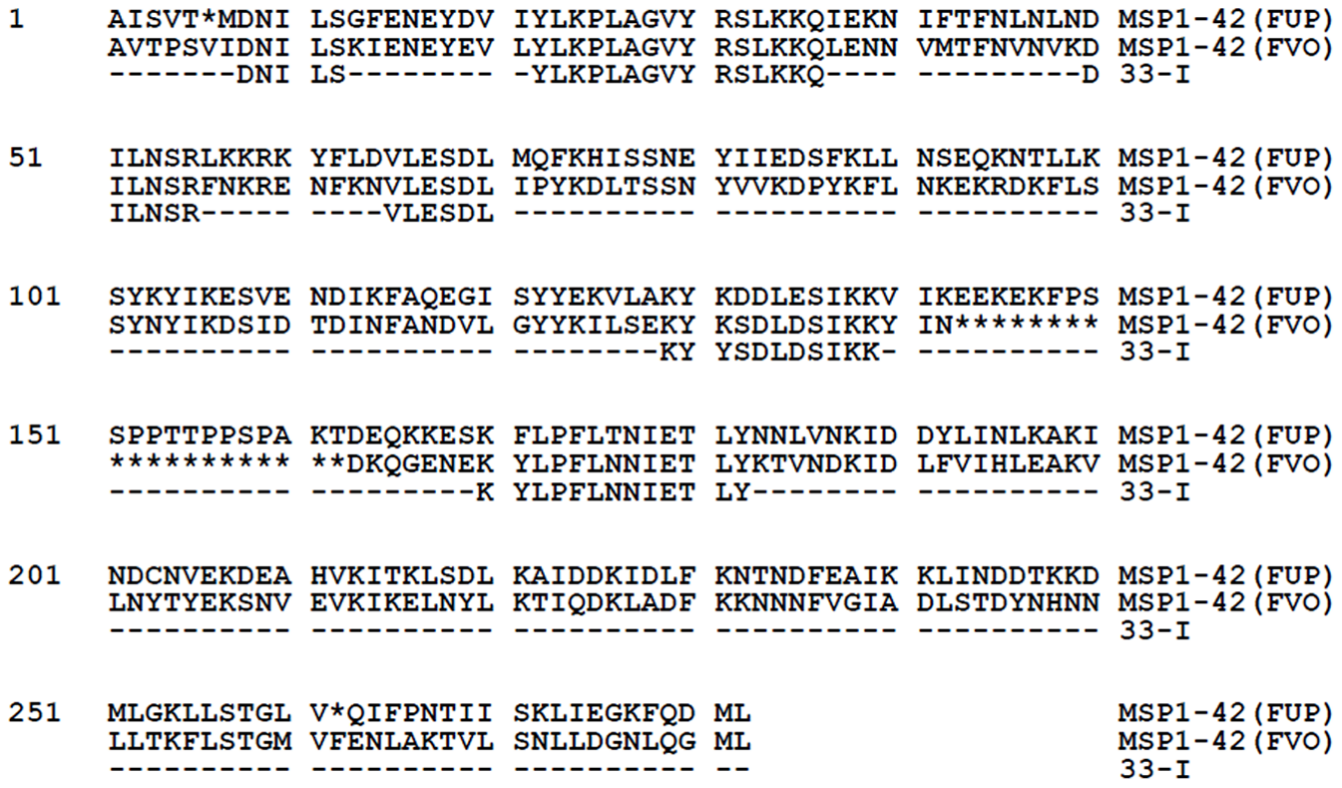
Aligned amino acid sequences of the truncated MSP1-42 protein, Construct 33-I, compared to full length MSP1-42. Sequences shown are for the N- terminal MSP1-33 fragment. All proteins contain the MSP1-19 fragment (not shown) at the C-terminal end. Amino acid sequences of the two dimorphic alleles of MSP1-42, represented by the FUP (MAD 20 equivalent) and the FVO (K1 equivalent) strains, are also shown.

Truncated MSP1-42 (Construct 33-I) was previously designed based on the *P. falciparum* FUP strain [28], was expressed in Drosophila S2 cells [29], and purified by affinity chromatography (Figure 1) [30]. Construct 33-I has been shown to maintain correct protein conformation and induce parasite growth inhibitory antibodies [28]. Full-length MSP1-42s representing both dimorphic alleles [31] [FUP strain (MAD20 allele) and FVO strain (KI allele)] were also expressed in Drosophila cells [29] and purified by affinity chromatography (Figure 1) [30].

### Prime-Boost Immunizations

Swiss Webster mice were immunized with different combinations of Construct 33-I and full-length MSP1-42 proteins. Immunization regimens are outlined in Table 1. A total of 36 SW mice were divided into six groups (*n* = 6 per group) and were primed via the i.p. route with either 10 ug/dose of truncated construct or full-length MSP1-42, emulsified in Complete Freund’s Adjuvant (CFA). Mice were then boosted 14 days later, with the same dose of the truncated construct or full-length MSP1-42 emulsified in Incomplete Freund’s Adjuvant (IFA) according to the regimens dictated in Table 1. Sera were obtained 21 days after the booster immunizations by tail vein bleeds, and mice were sacrificed for T cell analysis.

Cr: NIH(S)-nu/nu mice were divided into two immunization groups. Both groups were primed with 10 ug/dose of MSP1-42(FUP) emulsified in CFA via the i.p. route. Mice were then boosted 14 days later; one group received a 10 ug/dose of MSP1-42(FUP) and the other a 10 ug/dose of MSP1-42(FVO), emulsified in IFA. Sera were obtained 21 days later by tail vein bleeds.

### MSP1-specific Antibodies

Mouse sera were assayed for anti-MSP1 antibodies (MSP1-19) by direct binding ELISAs as previously described [32]. MSP1-19 antigen obtained from previous studies was used for coating ELISA plates [25]. The recombinant antigen was expressed in yeast and constructed based on the *P. falciparum* FUP strain [25]. Briefly, 96-well ELISA plates (Costar, Acton, MA) were coated with MSP1-19 at a concentration of 0.4 µg/mL. Plates were then blocked with 1% Bovine Serum Albumin (BSA) in Borate Buffered Saline (BBS). Test sera were serially diluted in 1% BSA/0.5% yeast extract/BBS and incubated for 60 minutes in the antigen-coated ELISA wells. Wells were washed seven times with High Salt Borate Buffered Saline (HSBBS) and incubated for 60 minutes with horseradish peroxidase conjugated anti-mouse IgG (gamma chain specific) antibodies (Kirkgaard and Perry Laboratories, Gaithersburg, MD) at a dilution of 1∶2000. Wells were subsequently washed as above and color development was made using the peroxidase substrates, H_2_O_2_ and 2.2′-azinobis (3-ethylbenzthiazolinesulfonic acid)/ABTS (Kirkgaard and Perry Laboratories, Gaithersburg, MD). Optical density (O.D.) was determined at 405 nm and endpoint titers were calculated and graphed using Sigma Plot 10®. End point titers were calculated using the serum dilutions that gave an O.D. of 0.2, which is greater than 4 fold of background O.D. absorbance obtained using normal mouse serum. Responders were defined as having an ELISA O.D. of >0.2 at the starting serum dilution of 1/50. Assays were performed using serum samples run in duplicates.

### ELISPOT Assays

ELISPOT assays of splenocytes harvested from immunized mice were performed according to previously described methods [33]. Briefly, 96-well PVDF plates (Millipore Inc., Bedford, MA) were coated with 10 ug/mL of the monoclonal antibody (mAb) against IFN-γ (R4-642) and 5 ug/mL of mAb against IL-4 (11B11) (BD Biosciences, San Diego, CA), and incubated overnight at room temperature. Plates were washed with Phosphate Buffered Saline (PBS) and blocked with 10% fetal bovine serum in DMEM for 60 minutes. Mouse spleens were harvested and single cell suspensions of splenocytes were prepared as previously described [33]. Purified splenocytes were plated at 0.5×10^6^, 0.25×10^6^, and 0.125×10^6^ cells per well and subunit recombinant protein (4 ug/mL) was added to each well as the stimulating antigen. Positive control wells were incubated with 5 ng/mL of phorbol myristate acetate (PMA) and 1 ng/mL of ionomycin. Plates were incubated at 37°C in 5% CO_2_ for 48 hours. Wells were washed and incubated with biotinylated mAb against IFN-γ at 2 µg/mL (XMG1.2), or mAbs against IL-4 at 1 µg/mL (BVD6-24G2) (BD, Biosciences, San Diego, CA), followed by the addition of peroxidase conjugated streptavidin (Kirkgaard and Perry Laboratories, Gaithersburg, MD) at a dilution of 1∶800. Spots were developed with a solution consisting of 3,3′-diaminobenzidine tetrahydrochloride (DAB) (Sigma-Aldrich St. Louis, MO, 1 mg/mL) and 30% H_2_O_2_ (Sigma-Aldrich St. Louis, MO) and enumerated microscopically. Data were presented as spot-forming-units (SFU) per million isolated splenocytes. Assays were performed using splenocytes plated in triplicates.

### Rabbit Immunizations

New Zealand White rabbits were immunized with Construct 33-I formulated in Montanide ISA51 adjuvant in a previous study [28]. Briefly, 50 µg of antigen in 250 µl PBS was emulsified with an equal volume of ISA51 as per the manufacturer’s recommendations. The emulsion was injected, via the IM route, into the left and right thighs. A total of four immunizations were given at 4 weeks intervals and sera collected 21 days after the last immunization were used in parasite growth inhibition assays.

### In Vitro Parasite Growth Inhibition Assays

The ability of rabbit sera generated by immunization with Construct 33-I to inhibit parasite growth was determined using an *in vitro* assay [10], [25], [34], [35]. The source of anti-Construct 33-I sera were from a previous study [28]. The inhibition assay was performed using sorbitol synchronized parasite cultures (3D7 strain and FVO strain) as described [25]. Synchronized parasite cultures at a starting parasitemia of 0.2% and 0.8% hematocrit were incubated in 30% heat inactivated immune sera. Cultures were then incubated for 72 hours with periodic mixing. Parasitemia was then determined microscopically by Giemsa staining. The degree of parasite growth inhibition was determined by comparing the parasitemias of cultures incubated with pre-immune sera as previously described [25], [34], [35].

### Data Handling and Statistics

Sigma Plot 10® and GraphPadPrism 4® were used to calculate end point titers. The Mann-Whitney test (GraphPadPrism 4®) was used to determine significant differences in antibody titers amongst the different test groups. Titers were analyzed and compared between the responders of each test group. Fisher Exact Test (GraphPadPrism4®) was also used to analyze the responders in each vaccination group. Cytokine responses (ELISPOT) in mice of different immunization groups were analyzed by Mann-Whitney test (GraphPadPrism4®). A p<0.05 was considered statistically significant.

## Results

### Antibody Responses Induced by Prime/Boost Immunizations with Full-length MSP1-42 Proteins were Entirely T Cell Dependent

MSP1-19 specific antibody responses were measured in Nu/Nu mice primed with the full-length MSP1-42 (FUP) and then boosted with either the homologous MSP1-42 or the heterologous MSP1-42 (FVO) allele. No MSP1-19 specific antibodies were detected by ELISA in either groups of mice (data not shown), indicating that the antibody responses induced by the prime/boost immunization regimens in this study (Table 1) were entirely T cell dependent, and thereby allowing subsequent assessment of the effectiveness of T helper epitopes within Construct 33-I in inducing antibody responses in the context of the full length MSP1-42.

### Reciprocal Prime Boost Immunizations with the Truncated MSP1-42 Protein (Construct 33-I) and Full-length MSP1-42 Induced MSP1-19 Specific Antibodies

Outbred Swiss Webster mice were tested for their ability to respond to and mount an antibody response (IgG only) against MSP1-19 as an indicator of effectiveness of Construct 33-I serving as a priming and/or boosting antigen.

**Figure 2:**
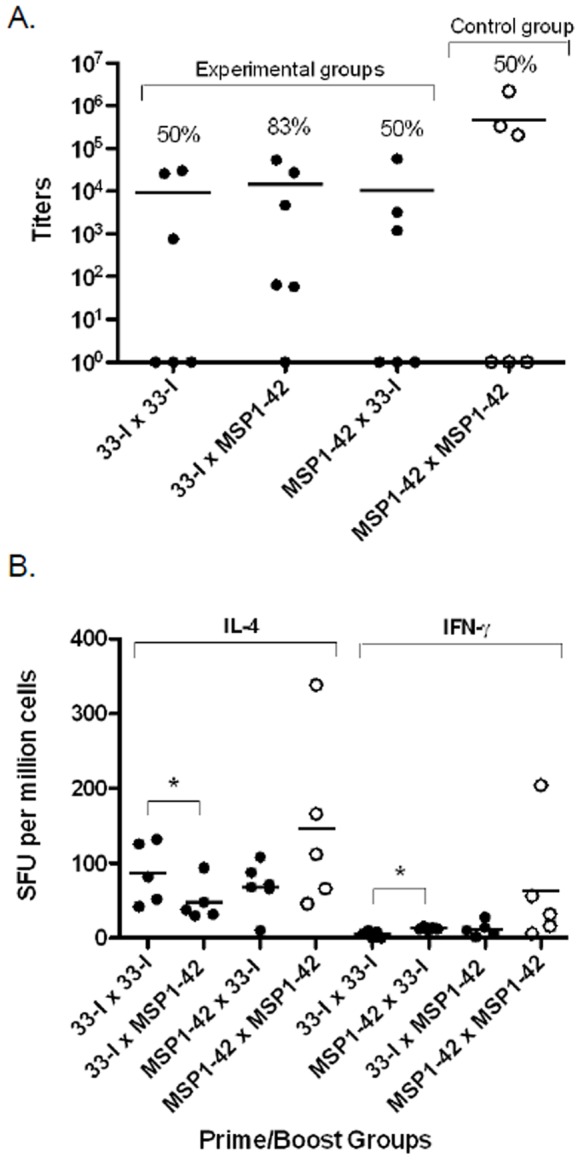
Antibody responses against MSP1-19 and antigen-specific T cell responses in reciprocal prime/boost immunizations in SW mice. Panel A, antibody titers of mice primed with Construct 33-I and boosted with MSP1-42; and antibody titers of mice primed with MSP1-42 and boosted with Construct 33-I. Filled circles represent the experimental groups with Construct 33-I. Open circles represent the control groups. The percent (%) of animals responding is shown above for each immunization group. Results of secondary bleeds are presented. Horizontal bars indicate mean antibody titers. Antibody titers were determined and analyzed for each individual responding animal. Panel B, antigen specific T cell responses as determined by ELISPOTs. IL-4/IFN-γ responses in mice primed with Construct 33-I, then boosted with MSP1-42; and IL-4/IFN-γ responses in mice primed with MSP1-42, then boosted with Construct 33-I. Asterisks indicate a significant difference between groups by the Mann-Whitney test; 33-I×33-I versus 33-I×MSP1-42 for IL-4 (*p = *0.0476) and IFN-γ (*p* = 0.0077). Filled circles represent the experimental groups with Construct 33-I. Open circles represent the control groups. The horizontal line in each bar indicates the mean SFU.

When Construct 33-I was used to prime mice and subsequently boosted with the full-length MSP1-42, the degree of responsiveness was higher than that observed when mice were primed and boosted exclusively with Construct 33-I or with the full-length MSP1-42 (Figure 2A). The reciprocal immunization regimen was also performed using Construct 33-I as the boosting antigen in mice previously primed with the full-length MSP1-42. Boosting with Construct 33-I induced the same response rate of 50% regardless of whether the full-length MSP1-42 or Construct 33-I was used as the priming antigen (Figure 2A, MSP1-42×33-I versus 33-I×33-I). There were no significant differences in the antibody titers of the responders among vaccination groups (Figure 2A).

As in previous studies of MSP1-42 constructs using SW mice [28], the range of antibody response within each mouse group was broad, as there were high, low and non- responders. The response rates were also low (50%) in the Control groups. The use of genetically heterogeneous mouse strain together with restricted use of only one prime and one boost regimen may have contributed to the broad range of antibody responses and lower response rate.

### Antigen Specific T Cell Responses in Reciprocal Prime Boost Immunizations with the Truncated and Full-length MSP1-42 Proteins

Splenocytes of immunized mice were stimulated *in vitro* with the same antigen used as the boosting immunogen and analyzed by IL-4 and IFN-γ ELISPOTS (Figure 2B). Priming and boosting with Construct 33-I induced a predominant IL-4 response with very low levels of IFN-γ production (Figure 2B, 33-I×33-I). Similar results were also observed in the reciprocal cross priming/boosting with Construct 33-I and full length MSP1-42 (Figure 2B, 33-I×MSP1-42; MSP1-42×33-I). This predominance of IL-4 over IFN-γ production was not due to the immunogenicity of Construct 33-I since it was also observed in mice primed and boosted with full-length MSP1-42 (Figure 2B, MSP1-42×MSP1-42).

### MSP1-42 Allelic Sequence Effects on Prime/Boost Immunizations with the Truncated and Full-length MSP1-42 Proteins

**Figure 3:**
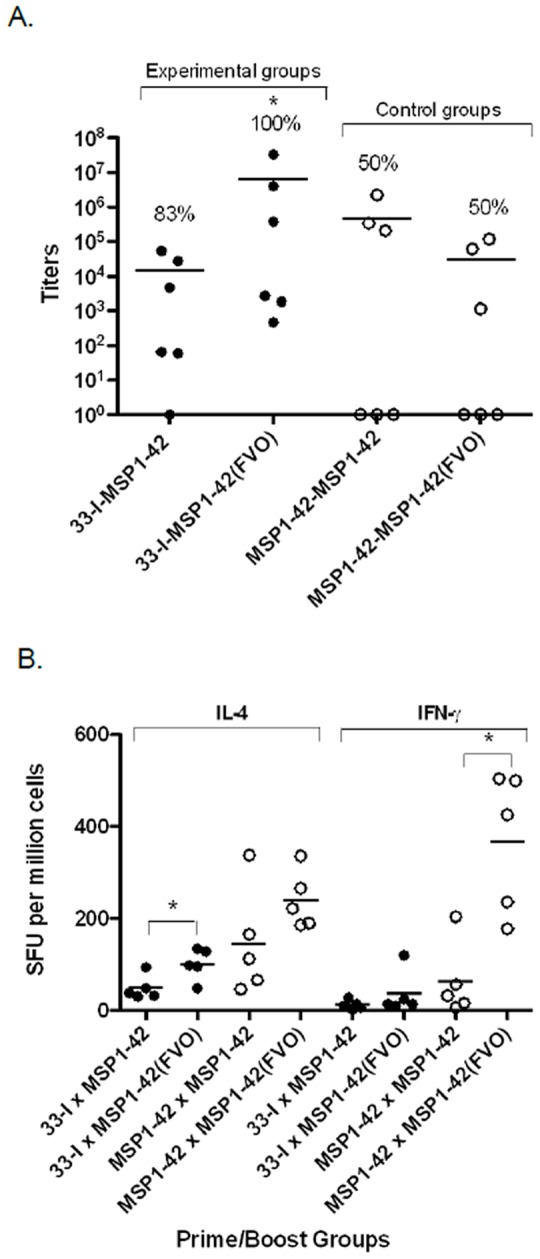
MSP1-42 allelic effects as determined by antibody and T cell responses, in prime/boost immunizations in SW mice. Panel A, antibody titers of mice primed with Construct 33-I and boosted with either the homologous (FUP) or heterologous (FVO) MSP1-42 allele (shown as filled circles). Open circles represent control groups primed and boosted with homologous or heterologous MSP1-42 alleles. Asterisk indicates a significant difference between groups by Mann-Whitney test; 33-I×MSP1-42 (FVO) versus MSP1-42×MSP1-42(FVO), p = 0.0452. The percent (%) of animals responding is shown above each immunization group. Horizontal bars indicate mean antibody titers. The response rates in the experimental groups are significantly different from the Control groups (Fisher Exact test; p = 0.0343). Panel B, antigen specific T cell response as determined by IL-4 and IFN-γ ELISPOTs. Immunization groups are the same as in Panel A and experimental groups are shown as filled circles and control groups are represented as open circles. Asterisks indicate a significant difference between groups by Mann-Whitney test; 33-I×MSP1-42 versus 33-I×MSP1-42(FVO) for IL-4 (*p = *0.0139) and between groups MSP1-42×MSP1-42 versus MSP1-42×MSP1-42(FVO) for IFN-γ (*p = *0.0079). The horizontal line in each bar indicates the mean SFU.

Secondary sera from Swiss Webster mice primed with Construct 33-I or full-length MSP1-42 and boosted with either the homologous or heterologous allele of the full-length MSP1-42 (Table 1) were tested for antibodies (IgG only) specific for MSP1-19 and antigen specific T cells. Construct 33-I primed mice had a greater number of responders (100%) when boosted with the heterologous MSP1-42 allele (FVO) than when boosted with the homologous MSP1-42 allele (83%) (Figure 3A). In comparison, mice primed with full-length MSP1-42 and boosted with either the homologous or heterologous MSP1-42 had lower percent responsiveness (50%) than Construct 33-I primed mice (Figure 3A). Mice primed with Construct 33-I and boosted with the heterologous MSP1-42 allele induced the highest mean antibody titers of all immunization groups (Figure 3A), but significant difference in titers was only observed between 33-I×MSP1-42(FVO) and MSP1-42×MSP1-42(FVO) (Mann-Whitney test, p = 0.0452). The ability of vaccination groups 33-I×MSP1-42 and 33-I×MSP1-42(FVO) to induce higher response rates were found to be significant when compared to the control vaccination groups MSP1-42×MSP1-42 and MSP1-42×MSP1-42(FVO) (Fisher Exact Test, one sided p-value = 0.0343). T cell responses in each immunization group (Table 1) were also analyzed by ELISPOTs. In general, heterologous MSP1-42 was significantly more efficient in boosting T cell responses than the homologous MSP1-42 allele [Figure 3B, 33-I×MSP1-42 versus 33-I×MSP1-42 (FVO) *p* = 0.0476; and MSP1-42×MSP1-42 versus MSP1-42×MSP1-42 (FVO) *p* = 0.0079]. The control vaccination groups for antibody titers and T cell responses are the same in Figure 2 and 3 since experiments were performed simultaneously.

We previously showed that Construct 33-I induced potent parasite inhibitory antibodies [28]. However, we were unable to test the inhibitory activities of the mouse sera here due to the restricted use of only two injections in the immunization regimens (Table 1) to evaluate the effects of cross priming/boosting of two different antigens. Under these conditions, antibody titers were not high enough to exert an effect *in vitro*. Further rounds of immunization will make the results difficult to interpret since the animal would have received at least two injections of the same antigen and the increase of antibody titer/response could have been due to boosting of identical epitopes of the same antigen. To further gauge the ability of Construct 33-I to elicit allele or strain transcending responses, immune sera from rabbits previously immunized with Construct 33-I [28] were evaluated for in vitro growth inhibition of parasites carrying the homologous MSP1-33 allele (3D7 strain) or the heterologous allele (FVO strain). Table 2 shows that the degree of growth inhibition by the anti-Construct 33-I sera against the 3D7 parasites (homologous allele) was similar to that when assayed against the FVO parasites (heterologous allele).

**Table 2:** In vitro Parasite Growth Inhibition of Rabbit Antibodies Generated by Construct 33-I.

Rabbit Sera (4^th^ Bleeds)	% Parasite Growth Inhibition^a^	
Anti-Construct 33-I	3D7 strain	FVO strain
Rbt #1	88%	83%
Rbt #2	84%	74%
Rbt #3	71%	60%

a Mean of two growth inhibition assays.

## Discussion

Studies with rodent malarias have established the importance of T cells in the induction of protective immunity specific for MSP1 [36]. We previously showed that different T helper epitopes on the *P. falciparum* MSP1-33 are able to exert positive or negative influences on the development of anti-MSP1-19 antibody responses and the induction of parasite inhibitory antibodies [28]. In line with these established work, we showed here that the induction of MSP1-19 specific antibody response is absolutely T cell dependent, as Nu/Nu mice did not produce detectable levels of anti-MSP1-19 antibodies. The result is critical for the prime/boost immunization regimens described here (Table 1) since we can rule out any detectable anti-MSP1-19 antibodies due to the potential contributions of T-independent antibody responses.

We recently demonstrated that a truncated MSP1-42 protein, Construct 33-I, based on six conserved sequences of MSP1-33 fused in tandem to MSP1-19, is able to elicit broad immune responsiveness and induce high levels of parasite growth inhibitory antibodies [28]. Since Construct 33-I was produced by artificially truncating and fusing individual segments of amino acid sequences of MSP1-33 [28], it is important to evaluate its immunogenicity in the context of recognition by immune responses to the full-length native MSP1-42 in order to gauge the potential immunological effects when this vaccine is deployed in populations exposed to malaria. The absolute T cell dependency for the induction of anti-MSP1-19 antibody responses, as demonstrated above using Nu/Nu mice, further underscores the importance of determining the extent of cross-reactivities between Construct 33-I and MSP1-42 induced responses.

Ideally, a MSP1-42 based vaccine should be able to elicit protective responses both in naïve individuals traveling to areas of malaria transmission and in malaria exposed subjects residing in endemic areas. In this regard, our reciprocal prime/boost studies performed on outbred Swiss Webster mice, which are genetically heterogeneous and would allow for a more stringent requirement for immunogenicity, provided encouraging evidence that Construct 33-I may be effective in both scenarios. Construct 33-I primed mice were efficiently boosted by the full-length MSP1-42 (Figure 2A); producing similar levels of MSP1-19 specific antibodies and antigen specific T cell responses (Figure 2B) as mice both primed and boosted with the same full-length MSP1-42, or with the same Construct 33-I. The similarity of antibody titers among these prime/boost groups suggests that antigen processing of the artificially truncated Construct 33-I can generate T helper responses comparable to control groups immunized with the full-length MSP1-42. We also analyzed the MSP1-19 specific Ig sub-classes for each prime-boost group but no significant differences in their Ig subclass profiles were observed, nor did the IgG1 levels correlate with cytokine profiles (data not shown).

Additionally, it is important to point out that the degree of responsiveness in the Construct 33-I primed MSP1-42 boosted group was higher than all other groups (83% versus 50%). In the reciprocal scenario where Construct 33-I was used as a booster antigen, it was able to recognize previously primed immune responses to the full-length MSP1-42 by enhancing or boosting MSP1-19 specific antibody (Figure 2A) and T cell responses (Figure 2B). The extensive cross-reactivity observed in these reciprocal cross priming and boosting experiments strongly suggests that antigen processing of Construct 33-I and MSP1-42 generated common T helper epitope(s) effective for anti-MSP1-19 antibody responses. Since Construct 33-I possesses only conserved sequences of MSP1-33, at least one of these conserved sequences is immunogenic as a T helper epitope shared by MSP1-42.

Prime/boost immunizations with Construct 33-I and/or the full length MSP1-42 preferentially induced IL-4 production from antigen stimulated splenocytes, suggestive of a TH2 polarizing response. While the role of TH1/TH2 responses in MSP1-42 specific immunity has not been established, protective immunity induced by MSP1-42 is clearly antibody dependent, and would be compatible with a TH2 biased immune environment.

It has been well established that anti-MSP1-42 and anti-MSP1-19 antibodies have biological function, i.e. inhibition of in vitro parasite growth/invasion [19]. Unfortunately, the biological activities of the anti-MSP1-19 antibodies induced in the different prime/boost groups could not be determined because of the relatively low antibody titers produced by two immunization doses (prime+boost). We have previously shown that significant parasite inhibition by anti-MSP1 antibodies can only be induced by hyperimmunization (4X), which would result in high antibody titers and/or antibody affinity [37]–[39]. Although further rounds of immunizations given to the various prime/boost groups here would achieve higher titers and affinity, it will be very difficult to analyze the growth inhibition results obtained in relation to the efficacy of the T helper responses. This is because animals receiving more than two doses of antigens would inevitably be boosted with the same antigen twice or more. Under these circumstances, the induction of inhibitory antibodies would at least in part due to potential contribution by the boosting of identical T helper epitopes of the same antigen rather than solely due to the efficacy of boosting crossreactive T epitopes of Construct 33-I and MSP1-42. The main focus of the prime/boost mouse studies here is to determined, under the stringent conditions of restricted two-dose exposure, whether T epitopes artificially fused together from different regions of MSP1-33 forming the Construct 33-I can be recognized by T cells generated by the processing of the natural MSP1-42. In this regard, the observed extensive crossreactivity between Construct 33-I and MSP1-42 indicates that the conserved T epitopes of Construct 33-I are highly functional. The utility of these conserved epitopes in enhancing immunogenicity may be extended to multivalent vaccines based on merozoite surface antigens since MSP1 has been shown to form complexes with other merozoite surface proteins such as MSP-6 and MSP-7 [40]. It is however important to reiterate that the efficacy of the T epitopes of Construct 33-I to potentiate anti-MSP1-19 parasite growth inhibitory antibodies has been established in hyperimmunizations of rabbits [28].

The phenomenon of strain-specific immunity is a hindrance to malaria vaccine development, as natural infections can arise from genetically heterogeneous parasites. Vaccines currently in development are only effective at protecting against infection by a homologous parasite strain [41]–[43]. In this light, it is very encouraging that the immunogenicity of Construct 33-I was unaffected in a heterologous allelic prime/boost regimen using the full length MSP1-42 (FVO) (Figure 3). Although this is not surprising since Construct 33-I possesses only conserved MSP1-33 sequences, the fact that the two allelic forms of MSP1-33 (Figure 1, FUP and FVO) differ not only in the dimorphic allele sequences but also in the overall length of the MSP1-33 polypeptides would indicate that the processing and generation of the putative cross-reactive, conserved T epitope(s) is remarkably similar between the two alleles. The allele or strain transcending nature of Construct 33-I observed in these heterologous prime/boost responses is further augmented by the ability of anti-Construct 33-I sera to inhibit the *in vitro* growth of parasites carrying the heterologous MSP1-42 (FVO strain); and to the same degree as parasites carrying the homologous allele (3D7 strain)(Table 2). In earlier studies, we showed that anti-MSP1-42 antibodies can inhibit parasite growth in allele and strain transcending fashion [44] and native MSP1 can induce robust antibody responses under the same heterologous allelic prime/boost immunization regimens [31]. Here, Construct 33-I, with only short conserved MSP1-33 sequences fused to MSP1-19, was able to produce the same cross-reactive responses as native MSP1 or MSP1-42. A more recent study on the full length MSP1-42 vaccine has concluded that pre-existing allele-specific immunity affects or constrains MSP1-42 vaccine immunogenicity, suggesting that a successful vaccine must be able to boost any preceding strain-specific malarial immune response [45]. Our study here provides much more encouraging evidence that the strategy of engineering a MSP1 vaccine based primarily on conserved sequences of MSP1-42 can effectively mount a strain transcending response irrespective of the allele identity of the pre-existing MSP1-42 specific immunity. Moreover, the strain transcending response can also be boosted by subsequent exposure to MSP1-42 of either allele. Further evidence that Construct 33-I has better vaccine potential than the full length MSP1-42 comes from the finding that mice primed with Construct 33-I and boosted with either the homologous or the heterologous full length MSP1-42 had higher immune response rates, i.e. 83% and 100% respectively, than prime/boost regimens with both MSP1-42 (50%) (Figure 2A & 3A). Thus, a vaccine based on Construct 33-I may be more broadly effective than the full length MSP1-42 vaccine in immunized populations subsequently exposed to native MSP1-42 during natural exposures/infections. Our previous studies with Construct 33-I indicate that some of the conserved putative T epitopes of MSP1-33 may bind multiple HLA alleles [28]. We hypothesize that Construct 33-I and MSP1-42 share limited T epitopes capable of promiscuous HLA binding; and these epitopes are subdominant. Moreover, the hierarchies of dominance of these shared epitopes are also different between Construct 33-I and the two MSP1-42 alleles, such that their immunogenicity, and hence their ability to elicit broader responsiveness, is affected by the prime/boost regimen. Lastly, computer analysis of Construct 33-I indicates that it consists of seven putative T epitopes [28]. One of these epitopes, spanning MSP1-33 amino acid position #21–35, has been shown to be recognized by human PBMCs in antigen driven proliferation assays [22]. Whether the remaining epitopes contribute helper function for anti- MSP1-19 antibody production is currently under investigation. Additional constructs each with one of the putative T epitopes fused to MSP1-19 will be analyzed for antibody immunogenicity.

Although our prime/boost immunizations with the truncated MSP1-42 antigen (Construct 33-I) and full length MSP1-42 do not fully represent antigen exposure during live infections, they provide a well controlled model to test the reciprocal influence by these immunogens on immunogenicity, and their possible outcomes when deployed as vaccines in the field. It is crucial for a malaria vaccine to take into account the nature of exposure to malarial antigens prior to and post vaccination as it has been shown to affect the outcome of protective immunity [46]. As our cross prime/boost studies here provided insights into the immune responses following two sequential antigen exposures, follow up studies simulating the effects of multiple and sequential infections as pre- or post-vaccine exposures may uncover the full picture of the influence of natural malaria exposures on the immunogenicity of Construct 33-I. Within the context of limited antigen exposure/experience, our results provide very positive evidence to this extent in that the immunogenicity of Construct 33-I can be sustained and/or enhanced under different manners of exposure to native MSP1-42. In some exposure scenarios, Construct 33-I proved to be a more effective immunogen than the full length MSP1-42. This, together with the allele/strain transcending responses afforded by Construct 33-I, suggests that deployment of this candidate vaccine in malaria endemic areas may be effective.
